# Redox imbalance in urology: mechanistic insights into the development and progression of selected urological conditions

**DOI:** 10.1186/s13062-026-00885-2

**Published:** 2026-10-14

**Authors:** Nadine Gelbrich, Matthias B. Stope

**Affiliations:** 1 Department of Urology, University Hospital Greifswald, Ferdinand- Sauerbruch-Straße, 17475 Greifswald, Germany; 2 Division of Plasma Medicine and Medical Technology, Department of Women’s Health, University of Tübingen, Calwerstraße 7, Tübingen, 72076 Germany; 3 NMI Natural and Medical Sciences Institute at the University of Tübingen, Reutlingen, 72770 Germany

**Keywords:** Redox signaling, Oxidative stress biomarkers, Ferroptosis, Antioxidant defense mechanisms, Tumor microenvironment, Hypoxia signaling, Metabolic reprogramming

## Abstract

Reactive oxygen species (ROS) are key mediators of cellular signalling and play a vital role in regulating both physiological and pathological processes. In urologically relevant tissues, particularly the prostate, bladder and kidneys, redox processes influence vascular function, immune responses, cell proliferation, metabolic adaptations and programmed cell death. A disruption of redox homeostasis contributes significantly to the development and progression of urological diseases, including prostate cancer, urothelial carcinoma of the bladder and renal cell carcinoma. This review article examines the mechanisms underlying the transition from physiological redox regulation to pathological dysregulation, as well as their significance for tumourigenesis, the tumour microenvironment, therapeutic resistance and disease progression. Finally, diagnostic and therapeutic perspectives of redox-based strategies, including innovative approaches such as medical gas plasma, are discussed.

## Background

The redox state of a cell is a fundamental regulator of biological systems. Electron transfer reactions govern essential processes including energy metabolism, signal transduction, and cell fate determination. Reactive oxygen species (ROS) are continuously generated during cellular metabolism and serve critical roles as signaling molecules [1–3]. At the same time, their uncontrolled accumulation leads to structural damage affecting DNA, proteins, and lipids.

The key determinant is therefore not the presence of ROS per se, but their integration into a tightly regulated balance. This balance, referred to as redox homeostasis, defines whether ROS exert adaptive or deleterious effects [2, 3].

In urology, this balance is of particular relevance. Urological organs are characterized by high metabolic activity, hormonal regulation, fluctuating oxygen supply, and frequent exposure to inflammatory stimuli. These features render them especially susceptible to redox-dependent dysregulation. ROS thus occupy a central position at the interface between physiological function and pathological transformation.

## Reactive oxygen species: definition, formation, and cellular control

**Fig. 1:**
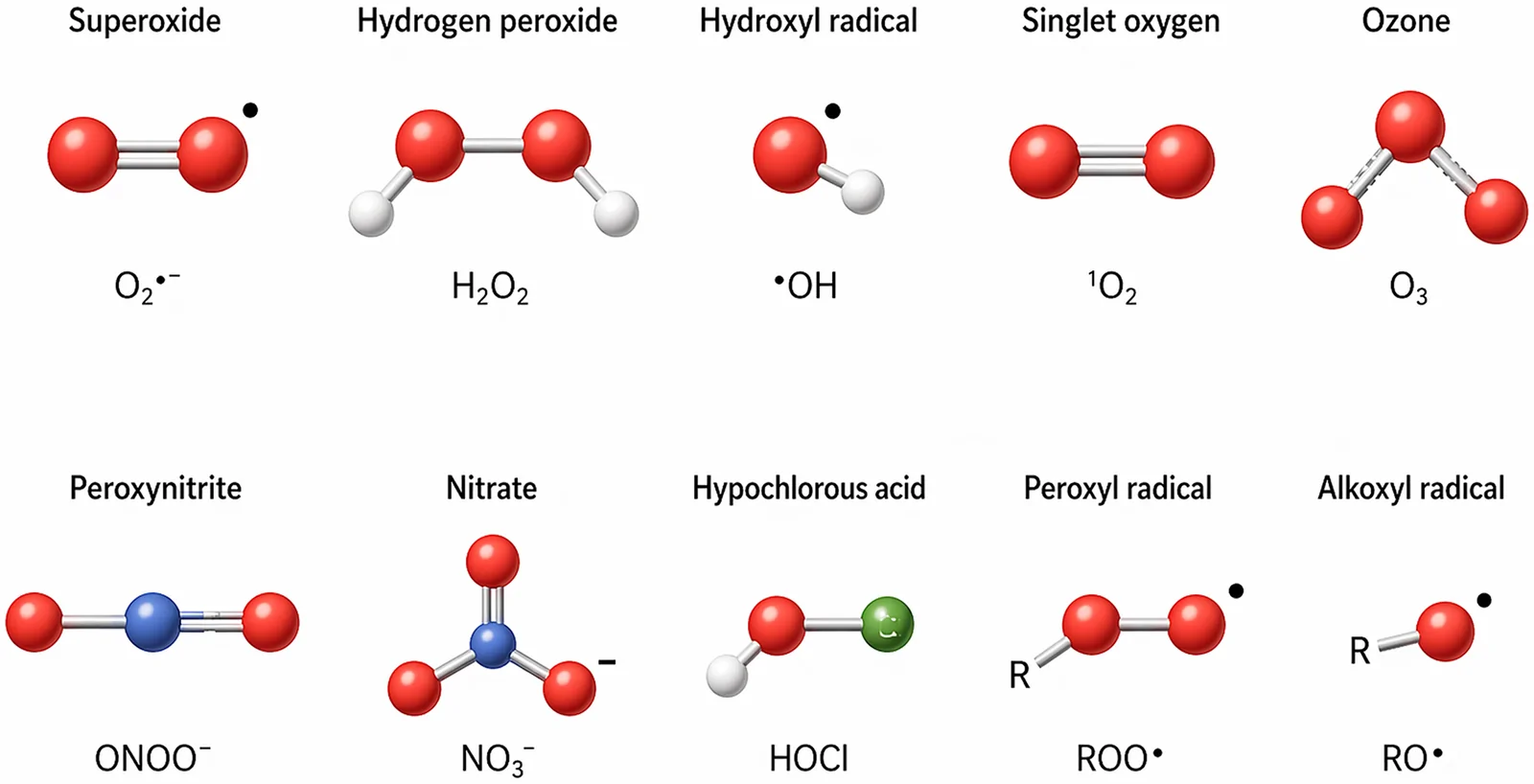
Representative molecular structures of selected ROS. Atoms are color-coded according to standard conventions, oxygen in red, hydrogen in white, and nitrogen in blue. Radical species are indicated by the presence of an unpaired electron. Each molecule is labeled with its full chemical name and corresponding molecular formula

ROS comprise a group of chemically reactive oxygen-derived molecules that include both radical and non-radical species (Fig. 1). Radical ROS contain unpaired electrons and exhibit high reactivity, such as superoxide anion and hydroxyl radical. In contrast, non-radical ROS such as hydrogen peroxide are more stable and diffusible, which allows them to function as signaling intermediates within and between cellular compartments [4–6].

ROS are continuously generated as part of aerobic metabolism. The mitochondrial electron transport chain represents a major source, where incomplete reduction of molecular oxygen results in superoxide formation, particularly at complexes I and III [4]. In addition to this constitutive production, ROS are generated in a regulated manner by dedicated enzymatic systems. NADPH oxidases produce ROS for host defense, signal transduction, and cellular adaptation [7]. Further sources include peroxisomal oxidases, oxidative protein folding processes in the endoplasmic reticulum, and metabolic enzymes such as xanthine oxidoreductase [5, 8, 9]. Under specific conditions, including cofactor depletion, nitric oxide synthases may become uncoupled and shift toward superoxide production, thereby increasing oxidative burden [9].

The generation of ROS is therefore not merely a by product of metabolism but an integral component of cellular signaling. At controlled concentrations, ROS act as second messengers by reversibly modifying redox-sensitive amino acid residues, particularly cysteine thiols, thereby regulating signaling pathways involved in proliferation, differentiation, immune responses, and stress adaptation [2, 5].

It is important to note that the biological effects of ROS are highly context-dependent. Whilst moderate concentrations of ROS can promote tumour cell proliferation, angiogenesis and cellular survival signals, excessive accumulation of ROS can lead to oxidative damage to DNA, proteins and lipids. If the cellular antioxidant capacity is exceeded in the process, apoptosis, ferroptosis or other forms of regulated cell death may be induced, thereby impairing cellular integrity and function and triggering tumour-suppressive effects [6, 10–14].

To balance ROS production and prevent uncontrolled oxidative injury, cells employ a complex network of antioxidant defense mechanisms. Superoxide dismutases catalyze the conversion of superoxide into hydrogen peroxide, which is subsequently detoxified by catalase and glutathione peroxidases [15–17]. Peroxiredoxins, together with the thioredoxin system, provide highly efficient and finely tuned buffering of hydrogen peroxide, particularly in the low concentration range relevant for signaling processes [18]. In parallel, non-enzymatic antioxidants such as glutathione contribute to maintaining intracellular redox balance and support enzymatic detoxification pathways.

Importantly, these systems do not eliminate ROS entirely. Instead, they maintain ROS within a controlled functional range that permits signaling while preventing toxicity. The continuous interplay between ROS generation and detoxification therefore represents a tightly regulated process rather than a simple defense mechanism.

## Redox homeostasis in urological tissues

Building on the molecular characteristics and cellular handling of ROS, redox homeostasis can be defined as the dynamic equilibrium between ROS generation, transformation, and detoxification. This equilibrium determines whether ROS primarily function as signaling mediators or as drivers of cellular damage.

In urological tissues, redox homeostasis is shaped by organ-specific physiological conditions. High metabolic activity, fluctuating oxygen availability, hormonal influences, and frequent exposure to inflammatory stimuli create a microenvironment in which ROS production and antioxidant capacity must be precisely coordinated. Under physiological conditions, this coordination enables ROS to regulate key processes such as vascular tone, epithelial function, immune surveillance, and cellular turnover.

At the cellular level, the biological impact of ROS is determined not only by their concentration but also by their spatial and temporal distribution. Compartmentalization plays a critical role, as ROS generated in mitochondria, peroxisomes, or at the plasma membrane can exert distinct signaling effects. Hydrogen peroxide, due to its relative stability and membrane permeability, acts as a central mediator that integrates localized ROS production into broader signaling networks [5].

Redox homeostasis is further controlled by the kinetic properties and localization of antioxidant systems. High-affinity enzymes such as peroxiredoxins and glutathione peroxidases efficiently buffer low ROS concentrations and enable selective signal transduction, whereas high-capacity systems such as catalase prevent excessive accumulation under conditions of increased oxidative load [15–18]. This hierarchical organization allows cells to maintain sensitivity to redox signals while preserving protection against oxidative damage.

Disruption of this balance results in oxidative stress, defined as a functional imbalance between ROS production and antioxidant defense. In urological tissues, such dysregulation may arise from chronic inflammation, ischemia, metabolic alterations, or environmental exposures. Importantly, oxidative stress is not solely characterized by increased ROS levels but by a failure of regulatory systems to maintain controlled redox signaling.

This shift from regulated redox signaling to dysregulated oxidative stress represents a critical transition from physiological adaptation to pathological processes. The following sections examine how this transition contributes to vascular dysfunction, inflammation, and tumorigenesis in urological diseases.

## From physiological regulation to pathological dysregulation

### Vascular function, hypoxia, and perfusion stress

ROS are integral components of vascular signaling and contribute to the regulation of endothelial function under physiological conditions. Controlled ROS production modulates calcium signaling, kinase activation, and transcriptional pathways that enable endothelial adaptation to shear stress and fluctuating perfusion demands [18–20]. In this context, ROS participate in maintaining vascular tone and barrier integrity.

A sustained increase in ROS levels, however, induces a shift toward endothelial dysfunction. Elevated ROS reduce nitric oxide bioavailability through direct scavenging and by promoting endothelial nitric oxide synthase uncoupling, thereby impairing vasodilation and promoting a pro-inflammatory and pro-thrombotic phenotype [20, 21]. These alterations compromise microvascular function and tissue perfusion.

The interaction between ROS and hypoxia further amplifies this process. ROS stabilize hypoxia-inducible factors (particularly HIF-1α) and thereby influence angiogenic signaling and metabolic adaptation under reduced oxygen availability [22]. Furthermore, ROS modulate various redox-sensitive signalling pathways, including PI3K/AKT and MAPK signal transduction, which play a key role in the regulation of cell survival, proliferation and stress response. At the molecular level, this occurs, among other things, via oxidative modifications of redox-sensitive cysteine residues in regulatory proteins. Examples of this include the reversible oxidation of the tumour suppressor PTEN with consequent activation of the PI3K/AKT signalling axis, as well as the oxidation of cysteine residues in the KEAP1 protein, thereby regulating the Nrf2-mediated antioxidant response [23–27]. In urological tissues, where perfusion can be highly dynamic, these mechanisms are particularly relevant. Experimental and clinical observations in the urinary bladder illustrate this relationship. Chronic bladder ischemia has been associated with oxidative stress, neuronal injury, and progressive functional impairment, providing a disease-specific example of how vascular dysfunction and ROS-mediated damage interact at the organ level [28].

### Inflammation and immune response

ROS play a dual role in the immune system as both effector molecules and signaling mediators. During the respiratory burst, phagocytic cells generate ROS to eliminate pathogens, while simultaneously regulating intracellular signaling pathways involved in cytokine production and immune activation [29, 30]. This controlled ROS generation is essential for host defense.

Under conditions of persistent stimulation, ROS contribute to chronic inflammation. Redox-sensitive signaling pathways, including NF-κB-associated networks, are activated by sustained ROS exposure and promote the transcription of pro-inflammatory mediators [31]. This establishes a self-reinforcing cycle in which inflammation enhances ROS production and ROS further sustain inflammatory signaling.

In urological tissues, which are frequently exposed to infectious, toxic, and mechanical stressors, this interplay is particularly relevant. Chronic inflammation associated with oxidative stress can lead to epithelial dysfunction and stromal remodeling. At the molecular level, prolonged ROS exposure induces oxidative DNA damage and impairs genomic stability, thereby linking inflammation to carcinogenic processes [32].

### Cell fate and regulated cell death

The biological effects of ROS on cellular fate are highly dependent on concentration, localization, and duration of exposure. At moderate concentrations, ROS act as signalling mediators that promote proliferation, differentiation and adaptive responses. These effects are mediated, amongst other things, via the activation of the PI3K/AKT and MAPK signalling pathways, which are key regulators of cell growth, cell cycle progression and survival signals. At the same time, ROS can influence the expression of numerous genes involved in the oxidative stress response, cell fate decisions and tumour progression through redox-sensitive modifications of proteins and transcription factors [24, 26, 33, 34].

This balance is particularly relevant in tumor biology. Cancer cells frequently maintain elevated ROS levels that support proliferative signaling but remain viable due to upregulated antioxidant defenses. This creates a narrow threshold between survival and cell death, rendering tumor cells vulnerable to further increases in oxidative stress [33].

Ferroptosis is a distinct, ROS-dependent form of programmed cell death characterised by iron-dependent lipid peroxidation [35, 36]. The accumulation of lipid hydroperoxides results from an imbalance between ROS formation and their detoxification, particularly when glutathione-dependent defence systems are impaired. Mechanistic studies suggest that ferroptosis involves both non-enzymatic lipid peroxidation and regulated enzymatic processes, including lipoxygenase-mediated pathways [37].

Key regulators of ferroptosis are glutathione peroxidase 4 (GPX4), the cystine/glutamate antiporter system Xc− with its functional subunit SLC7A11, and acyl-CoA synthetase long-chain family member 4 (ACSL4). GPX4 protects cells from ferroptosis by reducing toxic lipid hydroperoxides to non-toxic lipid alcohols using glutathione. The uptake of cystine via SLC7A11 is essential for glutathione synthesis and the maintenance of GPX4 activity. In contrast, ACSL4 promotes the incorporation of polyunsaturated fatty acids into membrane phospholipids, thereby increasing susceptibility to lipid peroxidation and ferroptosis [38].

In urological malignancies, ferroptosis has emerged as a relevant mechanism influencing tumour behaviour and response to therapy. Findings from urology-specific studies suggest that the modulation of ferroptotic signalling pathways could represent a potential strategy for selectively targeting tumour cells with altered redox states [39]. In particular, the targeted inhibition of cystine uptake via SLC7A11, the depletion of glutathione, or the pharmacological inhibition of GPX4 are currently being investigated as promising approaches to increase the sensitivity of tumour cells to oxidative stress and to exploit ferroptotic cell death programmes therapeutically [40].

## Redox dysregulation in urological diseases

### Benign prostatic hyperplasia

Benign prostatic hyperplasia (BPH) is increasingly recognized as a condition characterized by chronic redox imbalance rather than solely age-related tissue growth. Clinical and experimental studies demonstrate that oxidative stress in BPH is closely associated with persistent low-grade inflammation, stromal and epithelial proliferation, and altered growth signaling [41, 42].

Mechanistically, ROS contribute to pro-proliferative signaling pathways and tissue remodeling processes within the prostate. Experimental models have shown that oxidative stress can induce structural and functional changes consistent with hyperplasia, supporting a causal role of redox dysregulation in disease development [41]. These findings position BPH as a condition in which inflammation, hypoxia, and ROS-driven remodeling converge to shape tissue architecture.

### Prostate cancer

In prostate cancer, impaired redox regulation contributes to both the development and progression of the tumour. Elevated ROS levels promote oxidative DNA damage, genomic instability and epigenetic alterations, thereby facilitating malignant transformation [43]. At the same time, prostate cancer cells develop adaptive mechanisms that enable them to survive under oxidative stress.

A central component of this adaptation is the activation of antioxidant signalling pathways, including Nrf2-associated responses, which increase cellular resistance to oxidative damage [44]. Importantly, Nrf2 activation in prostate cancer is not exclusively controlled by ROS, but can also occur via ROS-independent mechanisms, reflecting a more comprehensive stress adaptation programme.

Furthermore, there is a close interaction between ROS and androgen receptor (AR) signal transduction, which plays a key role in the growth, survival and therapeutic response of prostate cancer cells. ROS can modulate AR activity directly or indirectly via redox-sensitive signalling pathways, whilst simultaneously activating proliferation-promoting signalling axes such as PI3K/AKT, MAPK and HIF-1α. At the molecular level, this occurs, among other things, through oxidative modifications of redox-sensitive proteins and transcription factors that influence the cellular stress response and tumour progression [45–49].

In advanced stages of the disease, particularly in castration-resistant prostate cancer (CRPC), redox signalling is closely linked to mechanisms of treatment resistance [50]. Chronic oxidative stress can promote genomic instability, DNA damage and epigenetic alterations, as well as activate adaptive signalling networks that enable the circumvention of androgen-dependent signal transduction. These include the activation of alternative growth and survival pathways, changes in AR expression, and the emergence of constitutively active AR variants. The development of castration resistance is therefore a multifactorial process in which, in addition to changes in the androgen receptor, redox-regulated signalling networks and the tumour microenvironment also contribute significantly to tumour progression and the limited efficacy of antiandrogen therapies [45, 51].

### Bladder cancer

In bladder cancer, the redox environment is strongly influenced by the urothelial interface, which is continuously exposed to environmental and urinary factors. These exposures contribute to increased oxidative stress, lipid peroxidation, and activation of signaling pathways involved in inflammation and angiogenesis [52].

Experimental evidence supports a direct role of ROS in urothelial carcinogenesis. Increased ROS production mediated by NADPH oxidases, such as NOX4, has been implicated in early transformation processes and may contribute to diagnostic applications in urothelial carcinoma [53]. In addition, ROS have been linked to the activation of signaling pathways that regulate hypoxia-responsive and angiogenic factors. However, these mechanisms are context-dependent and should be interpreted within specific experimental settings rather than as universally established pathways across all bladder cancers [52, 53].

In bladder cancer, molecular subtypes, particularly the luminal and basal classifications of muscle-invasive urothelial carcinoma, have high prognostic and therapeutic relevance. This classification is based predominantly on different gene expression profiles. Although the subtypes exhibit significant differences in terms of tumour biology, response to therapy and clinical course, no consistent differences regarding fundamental redox biological mechanisms have yet been demonstrated [54, 55]. ROS play an important role in established forms of treatment for bladder cancer. Many conventional treatment modalities, including radiotherapy and cytotoxic chemotherapy, exert their antitumour effect, at least in part, via increased intracellular ROS generation, which induces oxidative stress, DNA damage and ultimately the death of tumour cells. At the same time, ROS formation can also affect healthy tissue and thus contribute to the development of treatment-associated side effects [56–59].

Furthermore, ROS are involved in the mechanisms of action of immunotherapeutic approaches. In the context of intravesical BCG therapy, the uptake of BCG by immune and tumour cells induces the formation of reactive oxygen species, which promote the release of pro-inflammatory cytokines and the anti-tumour immune response via the activation of redox-sensitive signalling pathways, including NF-κB [60–63]. At the same time, ROS play a context-dependent dual role within the tumour microenvironment: whilst moderate concentrations can support the activation of immune cells, chronically elevated ROS levels can contribute to the functional exhaustion of antitumour immune cells and thereby limit the efficacy of immunotherapeutic approaches [64].

### Renal cell carcinoma

Renal cell carcinoma (RCC) represents a tumor entity in which redox regulation is closely integrated with metabolic adaptation. Clinical studies demonstrate increased oxidative stress markers, altered antioxidant capacity, and correlations between redox parameters and tumor stage, supporting the relevance of ROS in disease progression [65].

At the molecular level, redox signaling is tightly linked to metabolic reprogramming and hypoxia-associated pathways. The KEAP1- Nrf2 axis plays a key role in enabling tumor cells to tolerate oxidative stress and contributes to tumor survival and resistance to therapy [66]. In addition, ROS are embedded within a broader network of metabolic and hypoxic adaptations that characterize RCC biology [67].

### Shared mechanistic principles

**Fig. 2:**
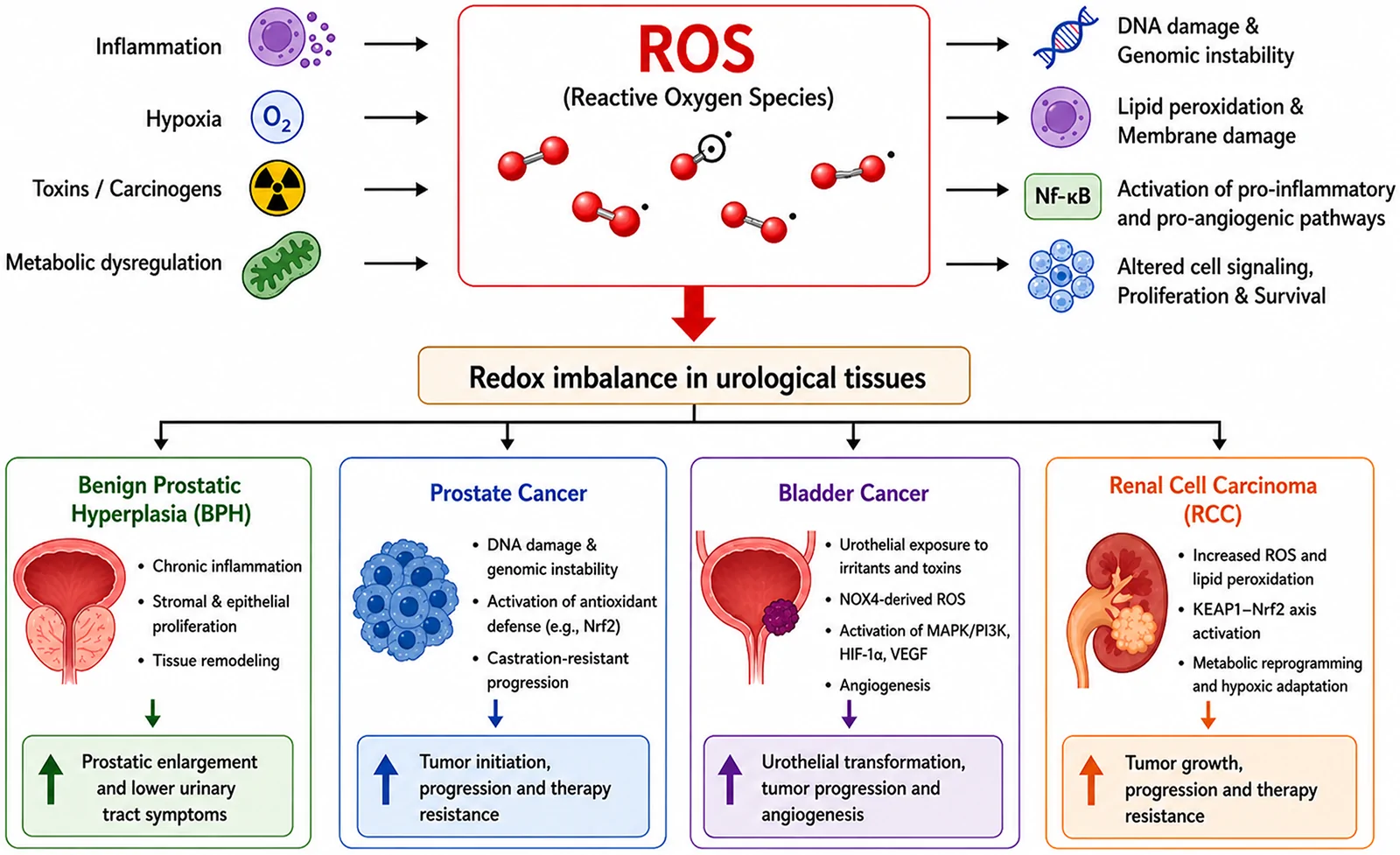
ROS-mediated effects in urological tissues and diseases. ROS arise from diverse sources, including inflammation, hypoxia, toxic exposures, and metabolic dysregulation, and induce molecular alterations such as DNA damage, lipid peroxidation, and activation of pro-inflammatory and pro-angiogenic signaling pathways. These processes disrupt redox homeostasis and promote disease-specific outcomes, including proliferative remodeling in benign prostatic hyperplasia, genomic instability and therapy resistance in prostate cancer, urothelial transformation and angiogenesis in bladder cancer, and metabolic adaptation in renal cell carcinoma

Across urological diseases, a common principle emerges in which pathological processes are not determined by absolute ROS levels alone but by the balance between ROS generation, antioxidant capacity, and tissue-specific context [68]. Disruption of this balance results in disease-specific patterns of redox dysregulation (Fig. 2).

These patterns differ between entities. In BPH, ROS are primarily associated with chronic inflammation and proliferative remodeling. In prostate cancer, they contribute to genomic instability and adaptive stress responses. In bladder cancer, ROS are linked to urothelial exposure and context-dependent signaling activation. In renal cell carcinoma, they are closely integrated with metabolic plasticity and antioxidant defense systems. This differentiation underscores that ROS function in urology is not uniform but shaped by organ-specific and disease-specific microenvironments.

## Diagnostic and therapeutic implications

Direct measurement of ROS is limited by their high reactivity, short half-life, and compartmentalization. Consequently, indirect markers of oxidative stress and global redox parameters are increasingly investigated. The most advanced clinical application is found in andrology, where oxidation reduction potential in seminal plasma is used as an adjunct diagnostic parameter in male infertility assessment. In other urological diseases, particularly uro-oncological entities, evidence for altered redox states is substantial, yet insufficient standardization and clinical validation currently limit routine diagnostic implementation. Overall, redox parameters may contribute to functional disease characterization in the future, but require methodological harmonization and prospective validation.

From a therapeutic perspective, the redox dependence of tumor cells represents a potential vulnerability. Strategies include increasing oxidative stress beyond tolerable thresholds or targeting antioxidant defense systems [28, 29]. Achieving tumor specificity while preserving normal tissue function remains a critical challenge.

Numerous established oncological therapies, including radiotherapy and various cytotoxic chemotherapeutic agents, already exploit the increased sensitivity of tumour cells to oxidative stress. Their anti-tumour effect is based, at least in part, on enhanced intracellular ROS generation, which induces DNA damage, mitochondrial dysfunction and ultimately cell death.

**Fig. 3:**
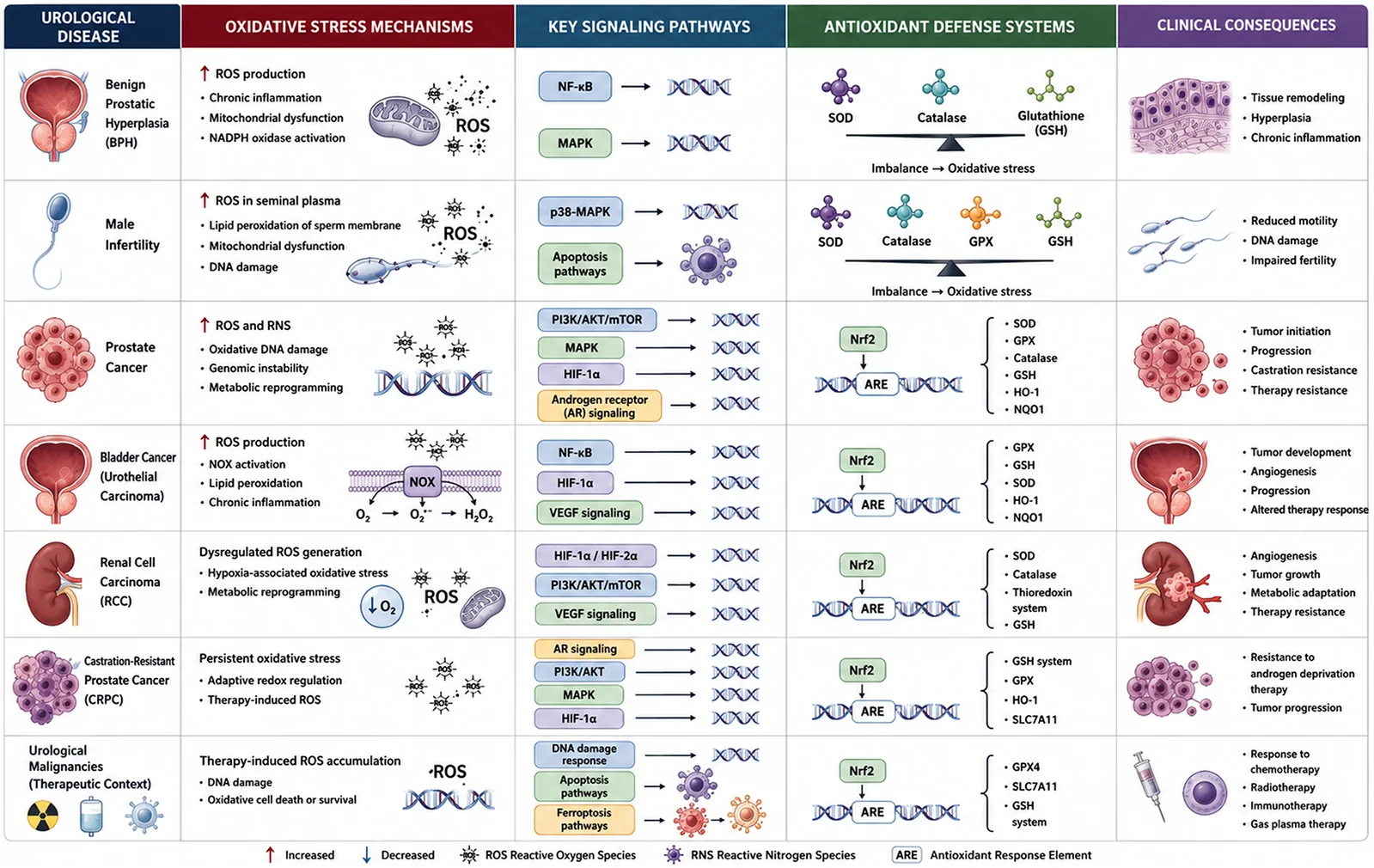
Characteristic Mechanisms of Oxidative Stress in Selected Urological Diseases. Schematic overview of major sources and biological consequences of reactive oxygen species (ROS) in selected urological diseases. Oxidative stress-induced redox imbalance contributes to disease-specific pathological processes through activation of redox-sensitive signaling pathways, modulation of antioxidant defense systems, and promotion of inflammation, angiogenesis, genomic instability, metabolic adaptation, and therapy resistance. The figure summarizes characteristic oxidative stress-related mechanisms and their clinical relevance in benign prostatic hyperplasia (BPH), prostate cancer, bladder cancer, and renal cell carcinoma (RCC)

In addition, targeted interventions in antioxidant defence mechanisms are increasingly coming into focus. These include the pharmacological inhibition of the Nrf2 signalling axis, the modulation of glutathione metabolism, and the inhibition of GPX4 or SLC7A11 to reduce cellular detoxification capacity and increase sensitivity to oxidative stress. Such approaches could be therapeutically relevant, particularly in tumours with pronounced redox adaptation (Fig. 3).

Another innovative approach is the use of medical gas plasma. This generates locally high concentrations of short- and long-lived ROS and reactive nitrogen species (RNS), which can specifically induce oxidative stress in tumour cells. Preclinical studies in various urological tumour models demonstrated inhibition of cell proliferation, the induction of apoptotic and ferroptotic signalling pathways, and selective tumour toxicity whilst largely sparing healthy tissue. Furthermore, there is evidence that gas plasma may also exert immunomodulatory effects and promote the anti-tumour immune response. Due to its local applicability and favourable safety profile, medical gas plasma represents a promising redox-based therapeutic approach, the clinical potential of which is currently being further investigated [69–71].

At the same time, significant clinical challenges remain. As oxidative signalling pathways are also essential for the function of healthy cells, a non-specific increase in ROS can lead to treatment-associated side effects and tissue damage. The development of tumour-specific strategies with sufficient selectivity is therefore an essential prerequisite for clinical application.

There is also growing interest in combinatorial therapeutic approaches, in which redox-modulating strategies are combined with chemotherapy, radiotherapy, targeted therapies or immunotherapies. Furthermore, the concept of synthetic lethality offers new therapeutic possibilities. Here, tumour-specific defects in DNA repair or antioxidant defence systems are specifically exploited to selectively eliminate tumour cells through additional redox stress, whilst healthy tissue is largely spared.

## Conclusion

ROS are integral components of urological disease mechanisms (Fig. 3). Their biological effects are governed by redox homeostasis, which serves as an overarching principle linking physiological regulation to pathological transformation. A mechanistic understanding of these processes provides a foundation for novel diagnostic and therapeutic approaches. Future research should focus on defining disease-specific redox architectures and translating these insights into clinically applicable strategies.

## Data Availability

No datasets were generated or analysed during the current study.

## Abbreviations

AR: Androgen receptor
BPH: Benign prostatic hyperplasia
DNA: Deoxyribonucleic acid
Fig.: Figure
GPX: Glutathione peroxidase
GSH: Glutathione
HIF: Hypoxia-inducible factor
KEAP1- Nrf2: Kelch-like ECH-associated protein 1 (Sensor)-Nuclear factor erythroid 2-related factor 2 (Transcription Factor)
MAPK: Mitogen-activated protein kinase
NADPH: Nicotinamide adenine dinucleotide phosphate
NF-κB: Nuclear factor kappa-light-chain-enhancer of activated B cells
NADPH: Nicotinamide adenine dinucleotide phosphate
NOX4: NADPH oxidase 4
Nrf2: Nuclear factor erythroid 2-related factor 2
PI3K: Phosphatidylinositol 3-kinase
PTEN: Phosphatase and Tensin homolog
RCC: Renal cell carcinoma
ROS: Reactive oxygen species
SLC7A11: Cystine/Glutamate antiporter
SOD: Superoxide dismutase
VEGF: Vascular endothelial growth factor
